# Evaluating the frequency of MLH-1 Loss in serrated polyps of colon: a single center study from Southern Iran 

**Published:** 2017

**Authors:** Bita Geramizadeh, Nooshin Dindar, Samirasadat Hasheminasab, Tahere Haeidari

**Affiliations:** 1 *Department of Pathology, Shiraz University of Medical Sciences, Shiraz, Iran*; 2 *Transplant Research Center, Shiraz University of Medical Sciences, Shiraz, Iran*

**Keywords:** Hyperplastic polyp, Sessile serrated adenoma, MLH-1 loss

## Abstract

**Aim:**

Evaluation of the role of MLH-1 loss in serrated polyps of colon in a population of South of Iran.

**Background:**

There has been extensive change in classification and pathogenesis of serrated polyps of the colon during the last 10 years. The new classification is mostly based on the knowledge of the molecular pathogenesis and the rate of progression to colorectal cancer in these types of polyps. One of the most common and early lesions in molecular pathogenesis of serrated colorectal cancer is loss of MLH-1.

**Patients and methods:**

In this study over 2 years (2012-13), 78 cases of colorectal polyps with serrated morphology resected in hospitals affiliated with Shiraz University of Medical Sciences were reclassified and investigated for MLH-1 loss by immunohistochemical method.

**Results:**

Out of the 78 colorectal polyps, 64 were classified as hyperplastic polyp (HP) and 14 as sessile serrated adenoma/polyp (SSA/P). There was no case of traditional serrated adenoma. Three cases of SSA/P located in right colon showed dysplasia. MLH-1 loss was detected only in these 3 cases. No case of HP or SSA/P without dysplasia showed MLH-1 loss.

**Conclusion:**

SSA/P is not a common serrated polyp in our population, i.e. it is much less common than HP. Although MLH-1 loss in serrated polyps of colon is overall rare, it is fairly common in dysplastic right sided SSA/P, which confirms this molecular change as an early event in serrated carcinogenesis.

## Introduction

 Until two decades ago, almost all colorectal polyps were divided into two main groups: hyperplastic polyp and adenoma (1). 

In the last few years, there used to be many confusing areas regarding nomenclature of serrated lesions of the colorectal area, but recently many of these confusing points seems to be resolving, mainly because of the discoveries in different molecular pathways in these serrated lesions. Despite the presence of serrated histology in different types of colorectal polyps, the molecular pathway and risk of malignant transformation are completely different (2). 

Microsatellite Instability (MSI) is commonly associated with defective DNA mismatch repair and has been reported in certain colorectal cancers (CRCs) that arise from the serrated pathway. Promoter methylation of hMLH1 has been reported in 28% to 72% of sessile serrated adenoma/polyps (SSA/P) (3). Epigenetic silencing of DNA mismatch repair gene (MLH1) by promoter methylation results in the MSI phenotype. This can be detected as decreased MLH1 expression in dysplastic areas of SSA/P (4). Therefore, in this study, we attempted to evaluate the incidence of MLH-1 loss in serrated polyps of the colon, i.e. hyperplastic polyps (HP), SSA/P, and traditional serrated adenoma (TSA) in our center as the largest referral center in southern Iran. 

## Methods

During the 2-year study period (2012-13), all 78 cases reported as HP, SSA/P or TSA were extracted from the pathology archives of hospitals affiliated with Shiraz University of Medical Sciences. All slides were reviewed by a GI pathologist (BG) and re-classified according to the last version of the WHO (2010, 4th edition) (5).

Demographic characteristics of the patients and size of the polyps were recorded from either the pathology or endoscopy reports.

Paraffin blocks of all cases were extracted from the pathology archives and prepared for immunohistochemistry for MLH-1. Routine immunohistochemistry staining was performed for MLH1 using the primary prediluted antibody (mouse monoclonal, Biocare). For this purpose, after deparaffininzation with xylol and blocking endogenous peroxidase with H2O2 and antigen retrieval with Tris EDTA, sections were incubated with primary Ab and then envision (k4061,Dako) and diaminobenzidine (DAB), they were counterstained with Hematoxylin. Negative nuclear staining was interpreted as MLH1 gene deletion. 

## Results

During the study period (2012-2013), there were 78 cases of serrated polyps in the colonoscopies performed in the hospitals affiliated with our center. There were 33 males and 45 female patients. The age range was 50-75 years (51.1 ± 14.7). Size of the polyps ranged from 0.2 to 1.2 cm (0.44 ± 0.18). The most common location of the polyps was right colon which was identified in 27 cases (34.6%), followed by rectum in 20 cases (25.6%).

Details of the characteristics of the serrated lesions including size, location, and demographic findings in these 78 cases are shown in table 1.

Among these 78 polyps, there were 64 (82.1%) cases of HP and 14 (17.9%) polyps met the histopathologic criteria of SSA/P. No case of TSA was found among these 78 cases during these 2 years. Among 14 cases of SSA/P, there were 3 cases (3.8%) with dysplasia. All three cases showed MLH-1 loss; i.e. there was no nuclear staining in immunohistochemistry by MLH-1 antibody. None of the cases of SSA/P without dysplasia showed MLH-1 loss. None of the cases with the morphology of HP showed dysplasia or MLH-1 loss. 

**Table 1:** Characteristics of different types of serrated lesions of 78 cases in the current study

		HP	SSA /Polyp
Total		64 (82%)	14 (18%)
Age		49.73 ± 14.36	57.35 ± 15.4
Sex		Male = 27 (42.2%) Female = 37 (57.8%)	Male = 6 (42.9%) Female = 8 (57.1%)
Size of tumor		0.4 ± 0.14	0.62 ± 0.24
Location	Right colon (Cecum and ascending colon)	19 (29.6%)	13 (92.8%)
	Hepatic flexure	-	1 (1.7%)
	Transverse colon	5 (7.8%)	-
	Splenic flexure	8 (12.5%)	
	Rectosigmoid	32 (50%)	-
Cytological dysplasia		-	3 (21.4%)
MLH-1 loss		-	3 (21.4%)

## Discussion

In the past, almost all of colorectal polyps used to be divided into two main groups of either hyperplastic polyp or adenoma. It means that all colon polyps were reported as either HP or adenoma (1). However, since the mid-1980s, there have been reports on HP lesions associated with neoplastic changes and malignant transformation. In 1996, Torlakovic et al. reported a group of serrated lesions with an abnormal architecture without cytological dysplasia, which they called “sessile serrated adenomas” (SSAs). In the last few years, there have been many confusing nomenclatures regarding serrated lesions of the colorectal area, many of which seem to be resolved in the most recent literature. The main reason for resolved confusion in the nomenclature of serrated lesions is the discoveries in molecular pathways and pathogenesis of different types of serrated lesions. Now, it is proved that despite the presence of similar serrated morphology in different types of colon and rectal polyps, the molecular pathway and risk of malignant transformations in each group are completely different, so accurate histologic diagnosis is crucial (2).

According to the most recent WHO classification in 2010, serrated polyps of the colon and rectum are a heterogeneous group of lesions including hyperplastic polyp (HP), sessile serrated adenoma/polyp (SSA/P) and traditional serrated adenoma (TSA) (5).

There are three most common genetic alterations in the progression of serrated lesions to colorectal cancers (serrated pathway of carcinogenesis) (6), one of which is abnormal silencing of certain genes by DNA methylation, which results in microsatellite unstable (MSI) cancers. (3). MSI can be present only in some microsatellites (MSI-low or MSI-L) or within many of them (MSI-high or MSI-H). Lesions that show no evidence of MSI are termed ‘microsatellite stable’ (MSS) (3,7). Epigenetic silencing of DNA mismatch repair gene (MLH1) by promoter methylation causes MSI-H phenotype. This can be detected as decreased MLH1 expression in dysplastic areas of SSA/P and as homogenously complete loss of expression in invasive MSI-H adenocarcinomas (8). It is noteworthy that MLH1 methylation starts early in SSA/Ps, and only reduced or complete loss of expression with extensive methylation is associated with dysplasia and progression of the serrated cancer (9). 

Based on the above mentioned reports, we performed this study to re-classify serrated lesions in our center and to find out the incidence of MLH-1 loss (by immunohistochemistry) in serrated lesions of colon. During 2 years, we found 78 polyps with serrated morphology, and after re-classification, 64 were categorized as HP, and 14 as SSA/P. We did not observe any case of TSA during the study period. According to our previous experience, the total incidence of serrated polyps (including HP, SSA/P and TSA) in the colon was about 30.3% (10). There has been another more recent study from Tehran which showed the incidence of SSA/P to be 3.6% (11). There has been only one study from Iran on the prevalence of MLH-1 loss in serrated polyps of the colon which reported 66% MLH-1 loss in 15 serrated polyps with no further classification (i.e. it includes all HPs, SSA, and TSAs) (12). According to previous studies, there are many controversial reports about MLH-1 loss in colorectal serrated lesions. In 2006 (8), a report from the USA showed higher MLH-1 loss in SSA/P compared to HP (60.3% vs. 32%); another report in 2008 from the USA on 48 HPs and 62 SSA/Ps showed lower prevalence of MLH-1 loss in SSA/P (16%) compared with HP (21%) (13). It seems that the difference in these two studies is most probably due to different nomenclature and histopathologic diagnosis. A more recent study from Korea showed 41% MLH-1 loss in 56 cases of SSA/P (15% without and 25% with dysplasia), and 24.4% MLH-1 loss in 45 cases of HP (14). The results of another study from Switzerland was very similar to our findings; i.e. there was no MLH-1 loss in 12 HPs. However, 37.5% of 16 cases with SSA/P showed MLH-1 loss (15). 

According to our experience, MLH-1 loss can be seen in cases of SSA/P, especially when there is dysplasia. This finding can have a practical implication of confirming dysplasia in suspicious cases by performing immunohistochemistry for MLH-1. 

In conclusion, the frequency of MLH-1 loss in serrated lesions has been very different in previous studies, most of which can be attributed to different nomenclature; however, it seems that this frequency is correlated with the existence of dysplasia in SSA/P lesions. Further multicenteric studies with more cases of SSA/P with and without dysplasia are necessary to confirm the results of this study.

## Conflict of interest

The authors declare that they have no conflict of interest.
